# Gallbladder mucinous carcinoma in a child with metachromatic leukodystrophy, case report and literature review

**DOI:** 10.1186/s12887-025-06500-z

**Published:** 2026-01-12

**Authors:** Qiang Bai, Bilin Xiong, Shan Pei, Jun Zhou, Jiantian Lu, Shuangqiong Pu, Li Li, Qinghua Xu

**Affiliations:** 1 Surgery Department of Kunming Children’s Hospital, Kunming, Yunnan China; 2 Kunming Key Laboratory of Children Infection and Immunity, Yunnan Province Clinical Research Center for Children’s Health and Disease, Yunnan Institute of Pediatrics, Kunming Children’s Hospital, Children’s Hospital Affiliated to Kunming Medical University, Yunnan Medical Center for Pediatric Diseases, Yunnan Key Laboratory of Children’sMajor Disease Research, Kunming, 650228 Yunnan China; 3 Dali University, Dali, Yunnan China; 4 Pathology Department of Kunming Children’s Hospital, Kunming, Yunnan China; 5 Radiology Department of Kunming Children’s Hospital, Kunming, Yunnan China; 6 Lijiang Women and Children’s Hospital, Lijiang, Yunnan China

**Keywords:** Mucinous adenocarcinoma, Gallbladder cancer, Metachromatic leukodystrophy

## Abstract

**Background:**

Metachromatic leukodystrophy (MLD) is an autosomal recessive genetic disorder caused by arylsulfatase A (ARSA) deficiency. Patients with MLD exhibit a high prevalence of gallbladder polyps and intestinal metaplasia. Although these precancerous lesions may progress to malignancy over time, gallbladder cancer is rarely diagnosed in children. Here, we present a case of gallbladder mucinous adenocarcinoma in a 5-year-old child, detailing its clinical, imaging, pathological, and genetic characteristics. To our knowledge, this is one of the earliest documented instances of pediatric gallbladder mucinous adenocarcinoma in MLD.

**Case presentation:**

A 5-year-old male patient was admitted with a recurrent intermittent epigastric pain for over six months. Imaging examinations revealed an enlarged gallbladder and thickened gallbladder wall. The patient underwent cholecystectomy, and histopathological analysis confirmed mucinous adenocarcinoma with low-grade intraepithelial neoplasia. A low frequency (6.31%) of somatic *KMT2C* gene mutations (c.2922A > T, p.L974F) was detected in the tumor tissue. Ten months later, the patient was readmitted due to the onset of abnormal neuropsychiatric behaviors and significant regression in both cognitive and motor function. Brain MRI revealed multiple, bilateral, symmetrical abnormal signals within the cerebral white matter. Whole exome sequencing (WES) identified a homozygous missense variant (c.640G > A; p.Ala214Thr) in the *ARSA* gene. A diagnosis of MLD was established bases on clinical and molecular findings.

**Conclusion:**

Pediatric gallbladder cancer is extremely rare. Although the role of low-frequency (6.31%) *KMT2C* variant in carcinogenesis is uncertain. When accompanied by neurological symptoms, there is high suspicion for an underlying genetic etiology. Radiological imaging plays a critical role in providing indications for the diagnosis of leukodystrophy. And genetic testing is helpful in detecting germline variations and somatic mutations in tumor tissues.

## Introduction

Gallbladder carcinoma (GBC) is a relatively rare, fatal malignancy with marked epidemiologic variations in ethnicities and geographic regions (3.7 to 27.3 per 10,000). The majority of gallbladder carcinomas (80–95%) are adenocarcinomas. These adenocarcinomas can be further classified into several histological subtypes, including tubular, papillary, mucinous, and signet-ring cell type [1]. Mucinous carcinoma of the gallbladder is extremely rare, accounting for approximately 2.5% of gallbladder cancers. The tumors typically manifest with acute cholecystitis-like symptoms. At diagnosis, they are generally large, advanced-stage lesions that demonstrate more aggressive biological behavior compared to conventional GBC [2]. The clinical presentation of GBC lacks distinct specificity, often mimicking benign conditions, which leads to misdiagnosis and inappropriate treatment. More than 50% of patients are diagnosed at an advanced stage. It is highly aggressive and represents one of the malignancies with the poorest prognosis [3, 4].

The development of gallbladder cancer has been linked to various genetic and environmental factors. Epidemiological studies have demonstrated significant associations between the development of GBC and exposure to various environmental factors, such as tobacco, alcohol, fried foods, and pesticides. Gallbladder-related diseases (gallstones and chronic infection) may also increase the risk of gallbladder cancer [5, 6]. The well-established risk factors for GBC, including porcelain gallbladder, gallbladder polyps, bile reflux, Mirizzi syndrome, bacterial infection and female hormones, may serve as significant predisposing factors for this disease [7–9]. Early studies have found that gene mutations associated with GBC, including alterations in oncogenes, tumor suppressor genes, DNA repair, cell cycle regulation, and epigenetic modifications [1, 4]. In addition, genes implicated in lipid metabolism and gallstone formation may also increase the risk of GBC [1, 10, 11].

Metachromatic leukodystrophy (MLD; OMIM #250100) is an autosomal recessive lysosomal storage disorder caused by pathogenic variants in the *ARSA* gene. The *ARSA* gene (OMIM #607574), located at chromosome 22q13.33, spans eight exons and encodes arylsulfatase A (ARSA)—a 509-amino acid lysosomal enzyme essential for sulfatide catabolism. Pathogenic variations in the *ARSA* gene result in arylsulfatase A deficiency, impairing sulfatide catabolism and leading to progressive accumulation of sulfatide within lysosomes. This primarily affects myelinating cells (Schwann cells and oligodendrocytes), ultimately causing widespread demyelination and progressive neurologic dysfunction [12, 13]. The severity of the disease and its onset time are related to the remaining activity of arylsulfatase A. Bi-allelic null variants (frameshift, nonsense) in the *ARSA* gene resulting in minimal residual activity, below 1% compared to controls, predicted an early onset and poor prognosis [14, 15]. In addition, the progressive accumulation of unmetabolized sulfatides within specialized secretory epithelia (such as the gallbladder and gastrointestinal mucosa) promotes precancerous lesions, including intestinal metaplasia, hyperplastic polyps, and low-grade dysplasia [16, 17]. Gallbladder precancerous lesions warrant particular vigilance due to their significant malignant potential. Multiple cases have been reported, among which only a few patients were diagnosed with gallbladder cancer when they were young [16, 18, 19].

We present a case of gallbladder mucinous adenocarcinoma initially manifesting with right upper quadrant (RUQ) pain. Following cholecystectomy, the patient exhibited progressive neurodevelopmental regression. The brain MRI scan indicates cerebral white matter dysplasia. Subsequent whole-exome sequencing (WES) identified a homozygous variant in the *ARSA* gene (c.640G > A; p.Ala214Thr), confirming the diagnosis of MLD.

## Case presentation

**Fig. 1:**
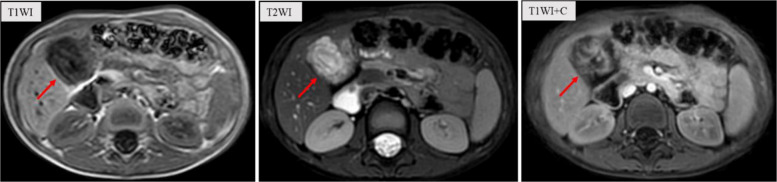
Abdominal MRI demonstrated an enlarged gallbladder

A 5-year-old male patient presented to our hospital with a recurrent, intermittent epigastric pain persisting for over six months, with no history of dietary indiscretion. Throughout the disease course, there had been no fever, vomiting, jaundice, abdominal distension, hematochezia, or other discomforts. Stool color remained normal, with no significant alterations in mental status, or body weight. Physical examination revealed tenderness in the right upper quadrant without rebound tenderness. The liver edge was palpable, 0.5 cm below the costal margin and 0.5 cm below the xiphoid process, with a smooth surface and sharp edge. Murphy's sign was positive. No visible gastrointestinal peristalsis or abdominal masses were detected. No abnormalities in the heart and lungs. An abdominal ultrasound performed at an external institution revealed gallbladder wall thickening (approximately 0.5 cm), accompanied by thickening of the surrounding tissues and enhanced echo. The echoes within the gallbladder are disordered and hypoechoic, with scattered patchy hyperechoic and anechoic areas visible among them (data not show). Abdominal magnetic resonance imaging (MRI) demonstrated an enlarged gallbladder with wall thickening and intraluminal signal abnormalities, accompanied by mild dilatation of the bilateral hepatic ducts, common hepatic duct, and mid-to-proximal common bile duct. The initial diagnosis is suspected to be biliary ascariasis and cholecystitis (Fig. 1). However, laboratory tests indicated that both the white blood cell count and eosinophil count were within the normal range. The concentrations of tumor markers alpha-fetoprotein (AFP) and carcinoembryonic antigen (CEA) were normal, and neuron-specific enolase (NSE) was slightly elevated 16.9 ng/ml (0–16.3 ng/ml). Liver enzymes were slightly elevated: ALT 63U/L (7–30 U/L), GGT 76 U/L (5–19 U/L), while amylase was increased (157 U/L) (0–96 U/L), with normal other biochemical profile. Positive EBV antibodies (IgG) indicating past infection. The remainder of the biochemical profile was unremarkable.

Following hospital admission, the patient received anti-infective, antispasmodic, and analgesic therapy, with subsequent improvement in paroxysmal abdominal pain. After completing preoperative evaluations, laparoscopic cholecystectomy was performed. Intraoperative findings included: normal liver morphology; marked edema and wall thickening of the distended gallbladder; no significant common bile duct dilation; and no pelvic fluid or purulent material. The gallbladder was completely excised. Upon sectioning, the gallbladder wall appeared grayish-white with a gelatinous consistency, containing minimal mucoid material but no bile, calculi, or parasites (data not shown).

**Fig. 2:**
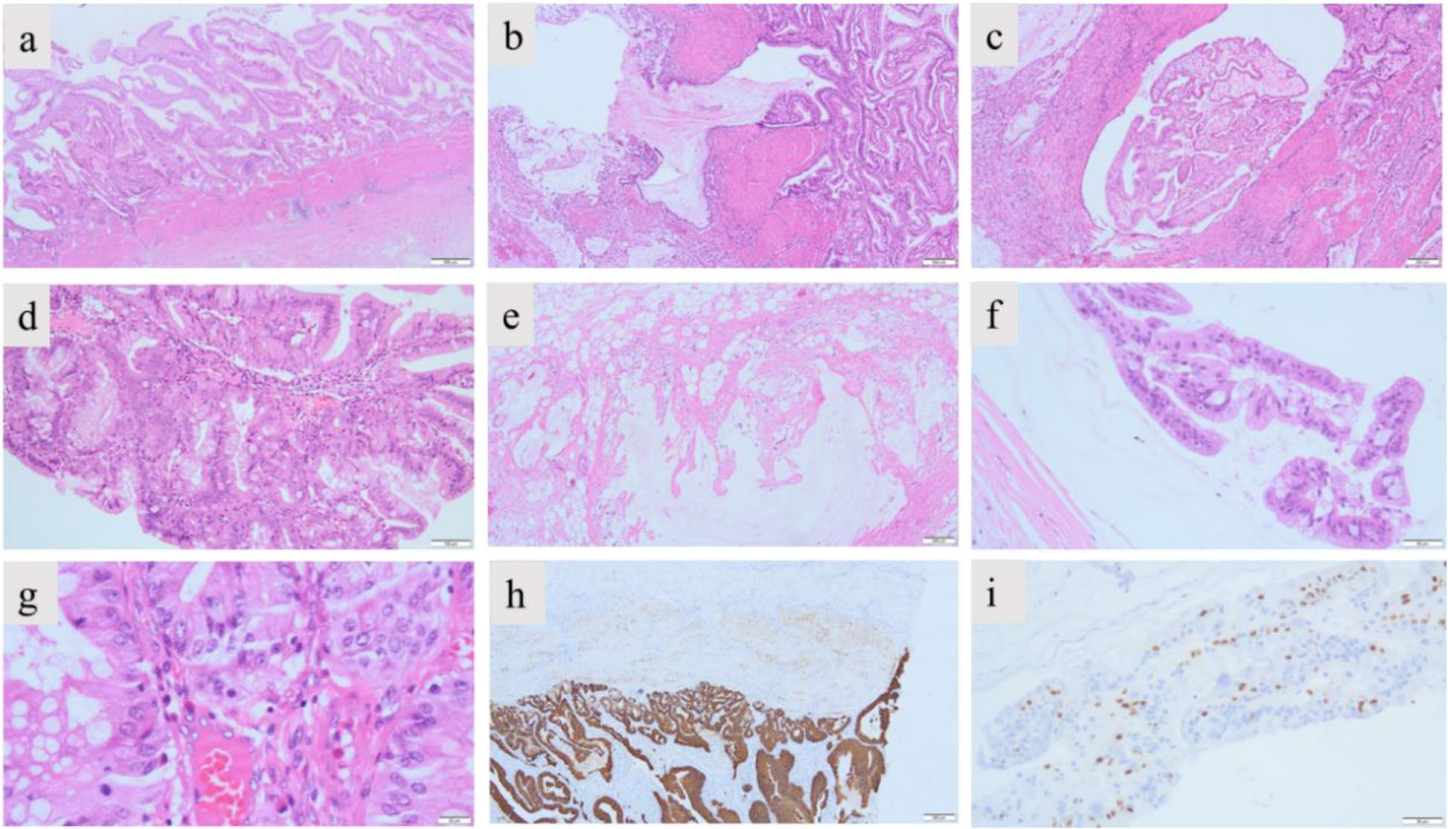
Histopathological examination of the resected gallbladder tissue **a** HE staining showed that the adenoma grew in a broad-based manner, presenting as polypoid and villous (magnification × 20); **b** and **c** HE staining showed that the tumor grows invasively and breaks through the muscular layer of the gallbladder wall (magnification × 40); **d** HE staining showed that the villous structure is lined with dysplastic glandular epithelium. The glandular epithelial cells are layered, crowded, partially sieve-like, with disordered cell polarity, and secretory and goblet cells can be seen (magnification × 100); **e** HE staining showed a large amount of mucus lake was in the serous muscular layer of the gallbladder wall (magnification × 40); **f** HE staining showed glandular epithelial fragments with atypia in the mucus lake (magnification × 200); **g** HE staining showed that the glandular epithelial cells are large in volume, crowded, with oval nuclei, and nucleolus and mitotic images can be seen (magnification × 400); **h** IHC demonstrated invasive growth of glandular epithelium from the mucosal layer into the muscularis propria (diffusely strong positive for CK8/18) (magnification × 40); **i** IHC showed that epithelial cell clusters within mucinous pools exhibited high proliferative activity (Ki67 positive) (magnification × 200)

Histopathological examination demonstrated villous-tubular gallbladder glands (with broad-based architecture) accompanied by low-grade glandular intraepithelial neoplasia (Fig. 2). Mucinous material and fragmented glandular epithelium were identified within the muscular layer (Fig. 2b, c). No neoplastic involvement was observed at the surgical resection margins. Immunohistochemical analysis demonstrated invasive growth of glandular epithelium from the mucosal layer into the muscularis propria (diffusely strong positive for CK8/18) (Fig. 2h). Epithelial cell clusters within mucinous pools exhibited high proliferative activity (Ki67 positive) (Fig. 2i). The diagnosis of gallbladder mucinous adenocarcinoma was established based on the extracellular mucin deposition constituting > 50% of tumor (Fig. 2e, f) [20]. In addition, genetic testing of tumor tissues detected a low proportion (6.31%) of somatic mutations in the *KMT2C* gene (c.2922A > T, p.L974F). The child recovered well after surgery, with no abdominal pain and no jaundane sclera.

**Fig. 3:**
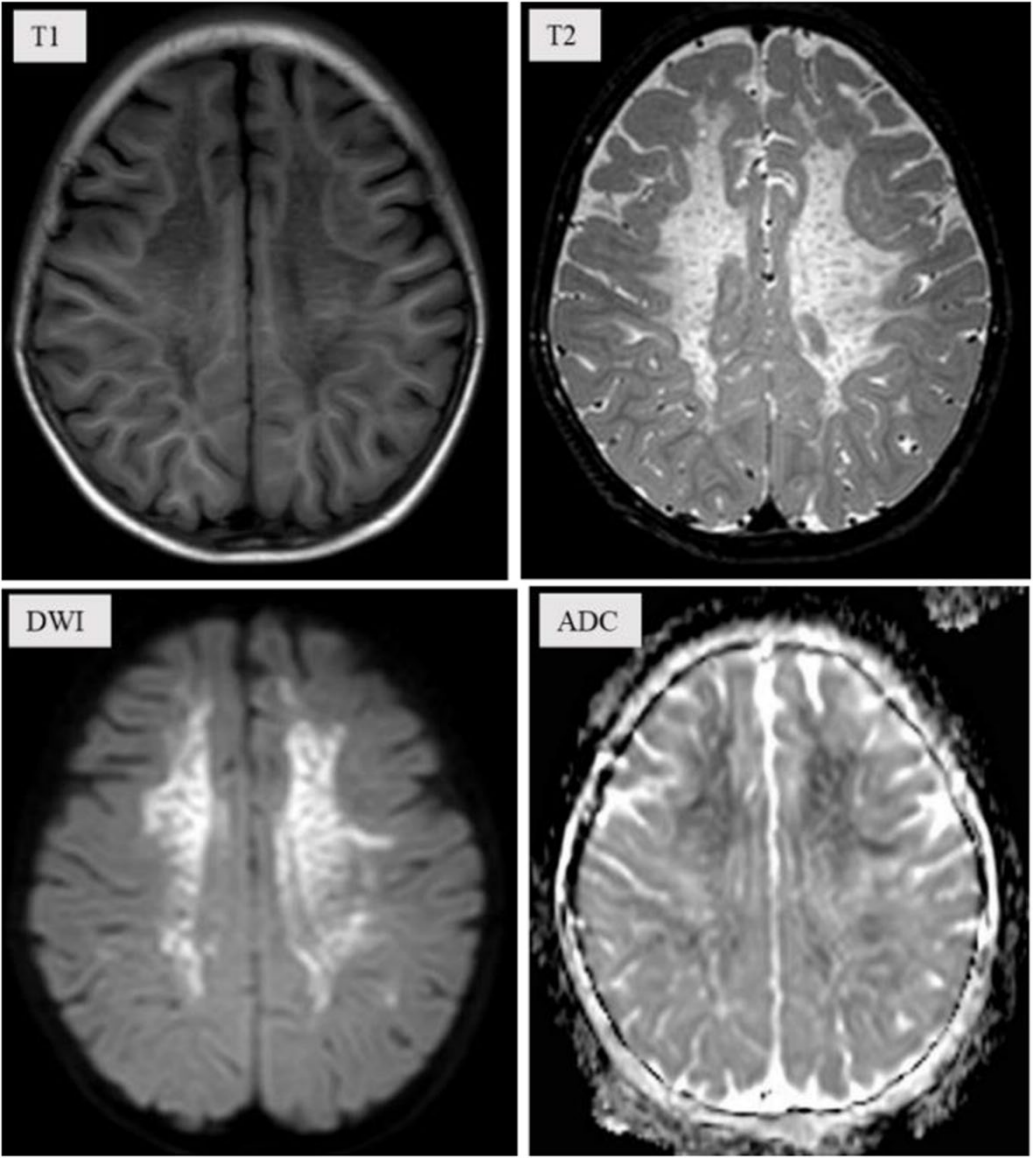
Brain MRI of showed "leopard skin sign" within the cerebral white matter

**Fig. 4:**
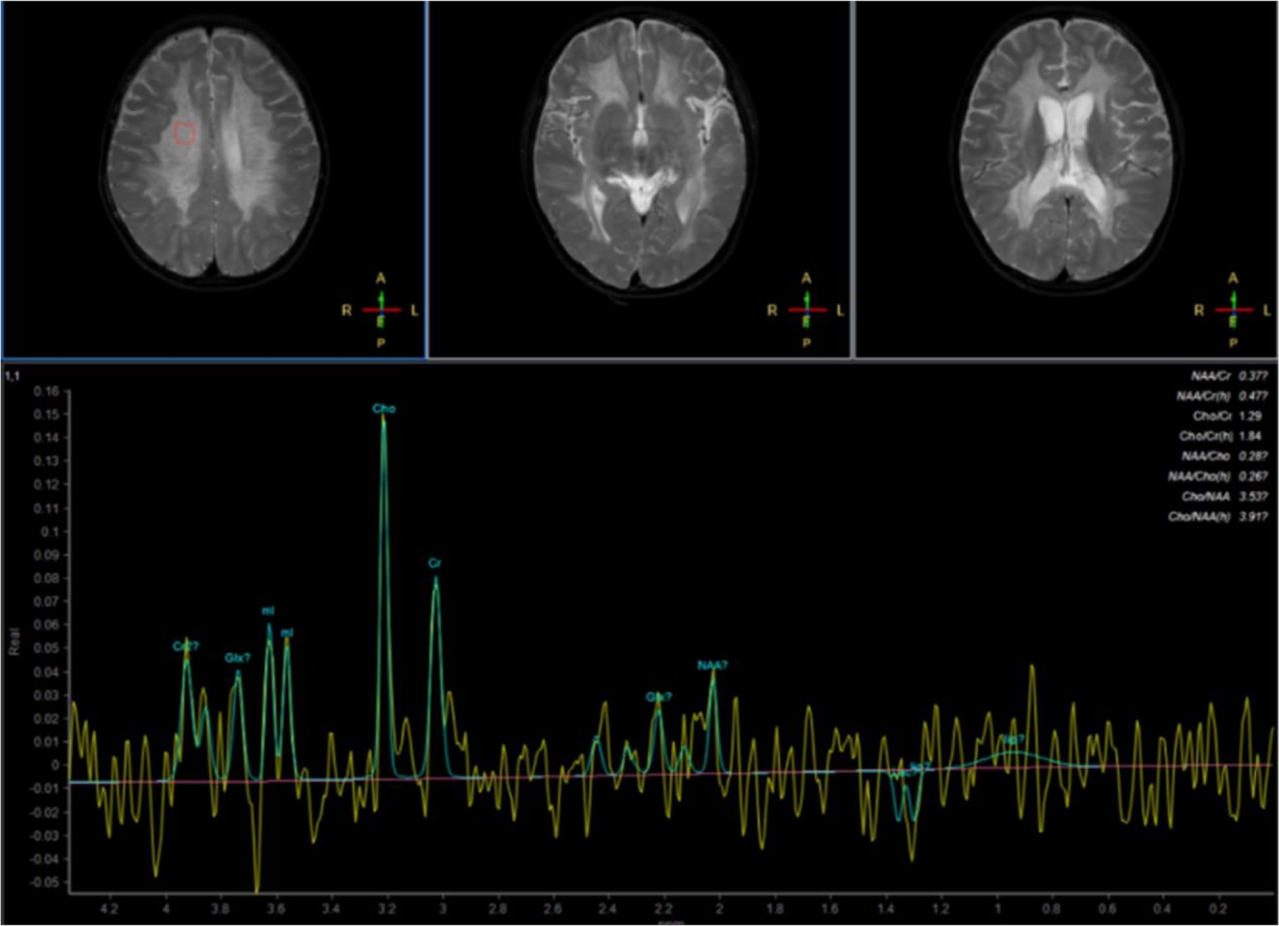
Brain MRS detected abnormal metabolic peaks in the cerebral white matter

**Fig. 5:**
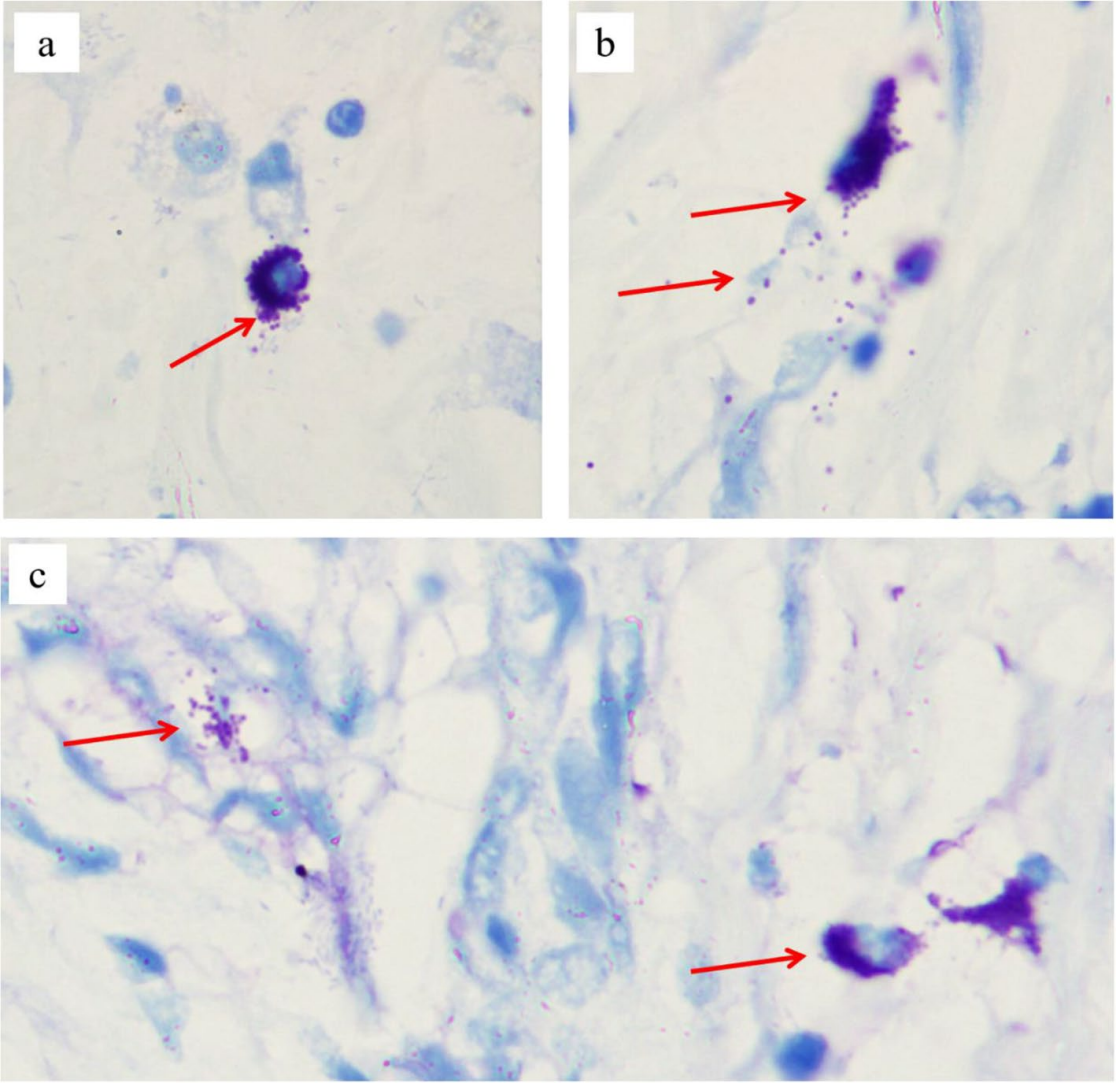
Toluidine blue staining performed on archived tissue sections. **a** The cytoplasm of scattered macrophages contains a large number of purple-red granules. **b** Cells were ruptured, resulting in the dispersal of purple-red granules extracellularly. **c** Purple-red granules are dispersed extracellularly, and the intercellular mucus also exhibits a purple-red coloration (magnification × 1000)

Ten months postoperatively, the patient was readmitted to the hospital following the onset of abnormal psychiatric behaviors accompanied by significant regression in both cognitive and motor function. According to parental reports, these behavioral changes emerged insidiously (about one year ago) without an identifiable precipitating cause. Manifestations included: purposeless hyperactivity (e.g., uncontrolled running), disruptive behaviors (e.g., discarding school supplies, retrieving and consuming waste materials), aggressive actions (biting), and inappropriate vocalizations (e.g., screaming). The patient demonstrated impaired social engagement (avoidance of peer interaction), loss of previously acquired play skills (inability to use toys appropriately), and failure to respond to simple commands. Additionally, the patient exhibited marked impairment in activities of daily living (ADLs), including an inability to self-feed, dress independently, or manage toileting. Concurrently, significant cognitive and motor regression was observed, characterized by: memory decline, apathy with reduced verbal output, dysarthria, unsteady gait with frequent falls, and deterioration of fine motor skills. MRI of the brain demonstrated numerous symmetrical, punctate signal abnormalities distributed throughout the white matter of both cerebral hemispheres, consistent with a classic 'leopard skin sign' pattern (Fig. 3). Magnetic resonance spectroscopy (MRS) demonstrated a reduced N-acetylaspartate (NAA) peak and an elevated choline (Cho) peak in the cerebral white matter (Fig. 4), indicating altered metabolic activity in this region. Laboratory analysis demonstrated that the patient's arylsulfatase A (ASA) activity was markedly reduced, measuring 10.13 nmol/17 h/mg—less than 10% of the mean value observed in healthy individuals. Whole exome sequencing (WES) identified homozygous likely pathogenic variations in the *ARSA* gene (c.640G > A, p.Ala214Thr). Toluidine blue staining performed on archived tissue sections (paraffin section) revealed numerous metachromatic (purple-red) sulfatide granules within foamy macrophages (Fig. 5), confirming sulfatide accumulation. Based on the patient's clinical presentation, neuroimaging findings, laboratory analyses, and genetic testing results, a definitive diagnosis of MLD was established. The clinical timeline of the patient is shown in Table 1.

**Table 1:** The clinical timeline of the patient

Timeline	Clinical symptoms and manifestations	Physical examination	Imaging examination and other tests	Diagnosis and treatment	Outcome
4.5 years old	Intermittent right upper abdominal pain			Treated at a local hospital, details unknown	The child continued to experience intermittent abdominal pain
4 years and 10 months old	Onset of abnormal mental and behavioral manifestations			The parents lacked vigilance and attentiveness, and no treatment was implemented	
5 years old	Intermittent upper abdominal pain	Tenderness in the right upper quadrant without rebound tenderness, Murphy’s sign (+)	Abdominal ultrasound and MRI: Enlarged gallbladder with a thickened wall	Anti-infective, antispasmodic, analgesic agents, cholecystectomy; Histopathological examination of the resected gallbladder: gallbladder mucinous adenocarcinoma	The child recovered well after surgery, with no abdominal pain and no jaundane sclera
5 years and 10 months old	Abnormal psychiatric behaviors and significant regression in both cognitive and motor function	Inattentiveness, slow response, sluggish movements, abdominal wall reflex (+ +), and knee reflex (+)	Brain MRI: Metachromatic leukodystrophy? WES: *ARSA* (c.640G > A, p.Ala214Thr)	Symptomatic treatment: vitamin B6, vitamin C, and other supportive agents	Given the patient's financial constraints, no further treatment was administered

## Discussion

### Gallbladder cancer and MLD

Gallbladder cancer is a relatively uncommon malignancy that predominantly affects elderly females. Histopathologically, the vast majority of cases (approximately 90%) are classified as adenocarcinomas [1]. Gallbladder mucinous adenocarcinoma represents an exceptionally rare histological subtype, comprising approximately 2.5% of all gallbladder adenocarcinomas [2]. Patients with MLD resulting from *ARSA* gene mutations exhibit a high prevalence of gallbladder polyps (27% ~ 39%) and intestinal metaplasia (75%) [16, 21]. Although these precancerous lesions may progress to malignancy over time, gallbladder cancer is rarely diagnosed in the early stages of life [16, 18, 22]. Compared with previously reported cases, the development of gallbladder cancer in this patient preceded the onset of neurological symptoms. This finding suggests that the natural course of the disease may not be uniform [21]. In 2021, Japanese researchers reported a case of gallbladder adenocarcinoma in a 5-year-old child with MLD. Pathological examination revealed high-grade dysplasia in cylindrical epithelial cells exhibiting papillary proliferation [19]. To our knowledge, this is the first report of gallbladder mucinous adenocarcinoma in a pediatric MLD patient.

### Genetic diagnosis

MLD is a rare autosomal recessive metabolic disorder caused by mutations of the *ARSA* gene. The patient was diagnosed with MLD based on the clinical manifestations, cranial MRI, and the identification of homozygous *ARSA* gene variants (c.640G > A, p.Ala214Thr), previously reported in a Chinese cohort [13]. In addition to a germline pathogenic variant in the *ARSA* gene, tumor tissue analysis revealed a low frequency of somatic mutations in the *KMT2C* gene. Could the early-onset gallbladder cancer in this case be attributable to an accelerated carcinogenesis process driven by somatic mutations? Previous studies have reported that in both humans and mice with arylsulfatase A (ARSA) deficiency, gallbladder epithelium and mucosal phagocytes contain abundant abnormal metachromatic sulfatide [16, 23]. In this study, foam cells containing abundant phagocytosed purple-red granules were identified in the patient’s paraffin-embedded tissue sections (Fig. 5). Although the use of non-frozen sections may have resulted in partial leaching of sulfatides, the histological findings remained consistent with sulfatide accumulation [23]. The accumulation of sulfatides and inflammatory stimulation may have induced polypoid hyperplasia and intestinal metaplasia of the gallbladder, which subsequently developed into carcinoma [16, 24].

Histone lysine N-methyltransferase 2 C (*KMT2C*) gene, mapped to chromosome 7q36.1, is a subunit of the mixed-lineage leukaemia (MLL) complex, which are methyltransferases specifically catalyze histone H3 on lysine 4 (H3K4) [25]. Mutations of *KMT2C* gene have been identified in a broad spectrum of malignancies, including liver cancer, gastric cancer, breast cancer, colorectal cancer, bladder cancer, lung adenocarcinoma, and acute myeloid leukemia [26]. The loss-of-function mutation of *KMT2C* observed in various cancers suggests a broader tumor-suppressor role for this gene [27–29]. *KMT2C* mutation in tumors may lead to epigenetic alterations in chromatin structure and reduce the transcriptional activity of tumor suppressor genes [26, 30]. Nevertheless, the relationship between *KMT2C* gene (OMIM # 606833) dysfunction and tumorigenesis remains unclear. Moreover, the frequency of the *KMT2C* variation detected in this study was very low (6.31%), and the degree of its functional impact was not significant.

### Imaging examination

The "leopard skin sign" is a characteristic neuroimaging manifestation of white matter dystrophy (Fig. 3), reported in MLD and other lysosomal storage disorders. These conditions are consistently associated with demyelinating pathology and varying degrees of psychomotor developmental abnormalities [31, 32]. Magnetic resonance spectroscopy (MRS) provides complementary biochemical information to the anatomical data obtained from conventional MRI, enabling a more comprehensive assessment of tissue composition and metabolic activity. In this case, MRS is characterized by a low N-acetylasparate (NAA) level and elevated Choline (Cho) (Fig. 4), which is slightly different from the previous reports [33].

### Strength and limitation

We characterized the complete clinical phenotype of a patient with MLD who developed early-onset gallbladder mucinous carcinoma—a rare complication that broadens the known phenotypic spectrum of the disease. The patient was initially diagnosed with rare gallbladder mucinous carcinoma. One year following cholecystectomy, regression in neuro-motor development was observed. Radiological imaging revealed white matter lesions. WES identified a homozygous variant in the *ARSA* gene, leading to a confirmed diagnosis of MLD. However, archived paraffin-embedded tissue specimens were utilized in this study. Given that sulfatides may leach out during tissue processing and sectioning, the histological findings may not fully represent the extent of sulfatide accumulation. Additionally, for financial reasons, the parents discontinued treatment and declined further follow-up, precluding longitudinal assessment of the patient's clinical outcome.

## Conclusions

Mutations in the *ARSA* gene result in the accumulation of sulfatides, which primarily cause demyelination within the nervous system. Additionally, sulfatide accumulation can affect lipid-secreting epitheliums such as the gallbladder, promoting precancerous lesions including intestinal metaplasia and polypoid hyperplasia. Somatic mutations may accelerate the process of carcinogenesis. When children present with rare malignant tumors alongside psychomotor regression, genetic metabolic disorders should be strongly suspected.

## Data Availability

The raw sequence data reported in this paper have been deposited in the Genome Sequence Archive (Genomics, Proteomics & Bioinformatics 2025) in National Genomics Data Center (Nucleic Acids Res 2025), China National Center for Bioinformation/Beijing Institute of Genomics, Chinese Academy of Sciences (GSA: CRA050059) that are publicly accessible at https://ngdc.cncb.ac.cn/gsa.
